# Influence of contrast medium on tophus detection using dual-energy CT: phantom study and clinical illustration

**DOI:** 10.1186/s41747-023-00348-7

**Published:** 2023-07-24

**Authors:** Maximilian Kotlyarov, Jürgen Mews, Sevtap Tugce Ulas, Katharina Ziegeler, Bernd Hamm, Torsten Diekhoff

**Affiliations:** 1 Department of Radiology, Charité — Universitätsmedizin Berlin, Campus Mitte, Humboldt-Universität Zu Berlin, Freie Universität Berlin, 10117 Berlin, Germany; 2 Canon Medical Systems, Europe BV, Zoetermeer, Netherlands

**Keywords:** Arthritis (gouty), Contrast media, Phantoms (imaging), Tomography (x-ray computed), Uric acid

## Abstract

**Background:**

To investigate the influence of iodinated contrast medium (ICM) on detection of monosodium urate (MSU) with dual-energy computed tomography (DECT) in two types of phantoms and demonstrate an example patient for clinical illustration.

**Methods:**

Approval is by the institutional review board, and written informed consent was obtained. A grid-like and a biophantom with 25 suspensions containing different concentrations of ICM (0 to 2%) and MSU (0 to 50%) were prepared and scanned with sequential single-source DECT using established methodology. Ascending orders of tube currents were applied at 80 kVp (16.5 to 220.0 mAs) and 135 kVp (2.75 to 19.25 mAs). Volume and mass measurements were performed using clinical gout software (dual-energy decomposition analysis). Numbers of true-positive and false-positive MSU detections were recorded and compared for different ICM concentrations. We demonstrate a patient with gouty arthritis for clinical illustration.

**Results:**

Effects of ICM on MSU detection varied with the amount of iodine. Lower ICM concentrations (0.25 and 0.50%) improved detection of small uric acid concentrations of 35 to 45% in comparison to scans without ICM. However, high ICM concentrations (1 and 2%) almost completely precluded MSU detection for all MSU concentrations investigated. In a patient with gouty arthritis, tophi in the wrist were only detected after intravenous ICM administration.

**Conclusions:**

Exploring multimodal DECT for arthritis imaging, enhancement of ICM influences tophus detection. It can help in visualizing previously undetected MSU depositions but, with too strong enhancement, also obscure tophi.

**Relevance statement:**

Use of iodinated contrast media in dual-energy CT might help in visualizing previously undetected uric acid depositions but, with too strong enhancement, obscure gouty tophi.

**Key points:**

• Iodine significantly influences the uric acid crystal detection in systematic phantom studies.

• Lower iodine concentrations improved detection of low and medium uric acid concentrations.

• High concentrations of iodine hampered detection of all uric acid concentrations.

**Graphical abstract:**

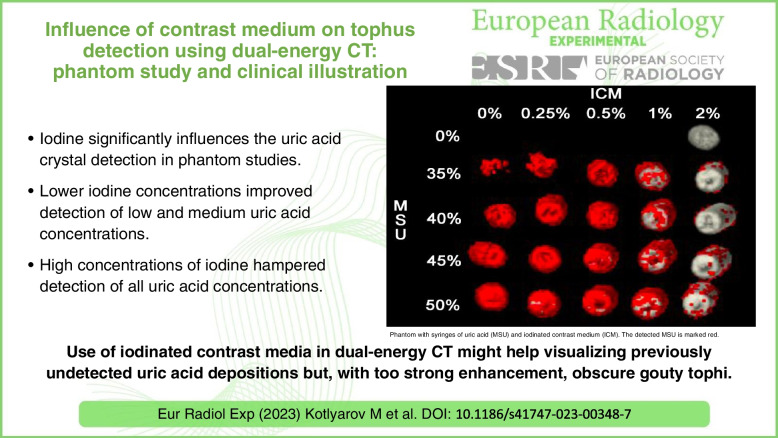

## Background

Chronic gouty arthritis is a polyarticular inflammatory disease and can mimic symptoms of other arthritic conditions such as rheumatoid or psoriatic arthritis. The hallmark of advanced gout is the tophus, which is a chronic foreign body granulomatous inflammatory response to the deposition of monosodium urate (MSU) crystals. Microscopically, a tophus consists of a crystalline core of MSU, a surrounding highly cellular corona zone, and an outer fibrovascular zone [1]. While rarely necessary to diagnose acute gouty arthritis, imaging workup is required for correct identification of chronic and atypical forms to derive the best treatment options [2, 3]. Dual-energy computed tomography (DECT) can detect MSU by measuring attenuation properties at different x-ray energies and applying two-material decomposition analysis. It is a highly specific imaging modality that is especially useful for detecting gouty tophi in atypical localizations [4, 5]. Vendors offer different techniques to perform DECT, one of them being single-source DECT which creates dual-energy datasets on conventional single-source CT scanners by consecutive acquisition of two single-source volumes with different tube voltages [6].

A recent development is the use of multiparametric DECT as a noninvasive diagnostic tool for diagnostic workup of patients with unclear arthritis, allowing simultaneous detection of erosions, bone marrow edema, gouty tophi, and — when an iodinated contrast medium (ICM) is administered — even active soft tissue inflammation: iodine maps generated from DECT datasets can be used to detect and quantify higher perfusion in synovitis and other signs of musculoskeletal inflammation [7, 8]. A recent study has shown DECT iodine maps to be similar to magnetic resonance imaging (MRI) in diagnostic performance when arthrosonography is used as the standard of reference [9]. While accurate detection of MSU by unenhanced DECT is well investigated, data on whether gouty tophi could also be detected by contrast-enhanced DECT are sparse, and it is unclear if the use of ICM in arthritis imaging could hamper the detection of gouty tophi. Given the different characteristics of ICM and MSU in spectral imaging, caution is advisable when it comes to applying standard two-material decomposition algorithms, as the presence of iodine could impact the calculation of dual-energy gradients of tophus material. Since patients with unclear arthritis undergoing contrast-enhanced multiparametric DECT may also suffer from gout, currently an unenhanced DECT is performed routinely beforehand due to the unknown effect of ICM on the detection of MSU.

Therefore, the aim of this study was to systematically analyze whether, and if so, how the presence of ICM influences the detection of MSU in different *ex vivo* phantom settings.

## Methods

Approval is by the institutional review board (EA1/183/21), and written informed consent was obtained.

### Preparatory work

To get a better understanding of how much ICM is needed for the phantom design to simulate the contrast enhancement of inflammatory periarticular soft tissues and synovitis, we started by determining the required concentrations of ICM. To this end, region of interest density measurements were performed in CT scans from a group of twelve patients with arthritis of the hand investigated in a previous study with MRI as a reference [10]. To quantify contrast enhancement, measurements were placed in areas of synovitis and contrast-enhancing soft tissues around the arthritic joints of the patients in subtraction images of the dynamic contrast-enhanced CT scans at 80 kVp. Mean enhancement of inflammatory tissue was 91.67 Hounsfield units (49 to 160 HU). Based on these results and published data, we chose ICM concentrations of 0, 0.25, 0.5, 1, and 2% to achieve attenuation in this HU range [11].

### Phantom model

**Fig. 1:**
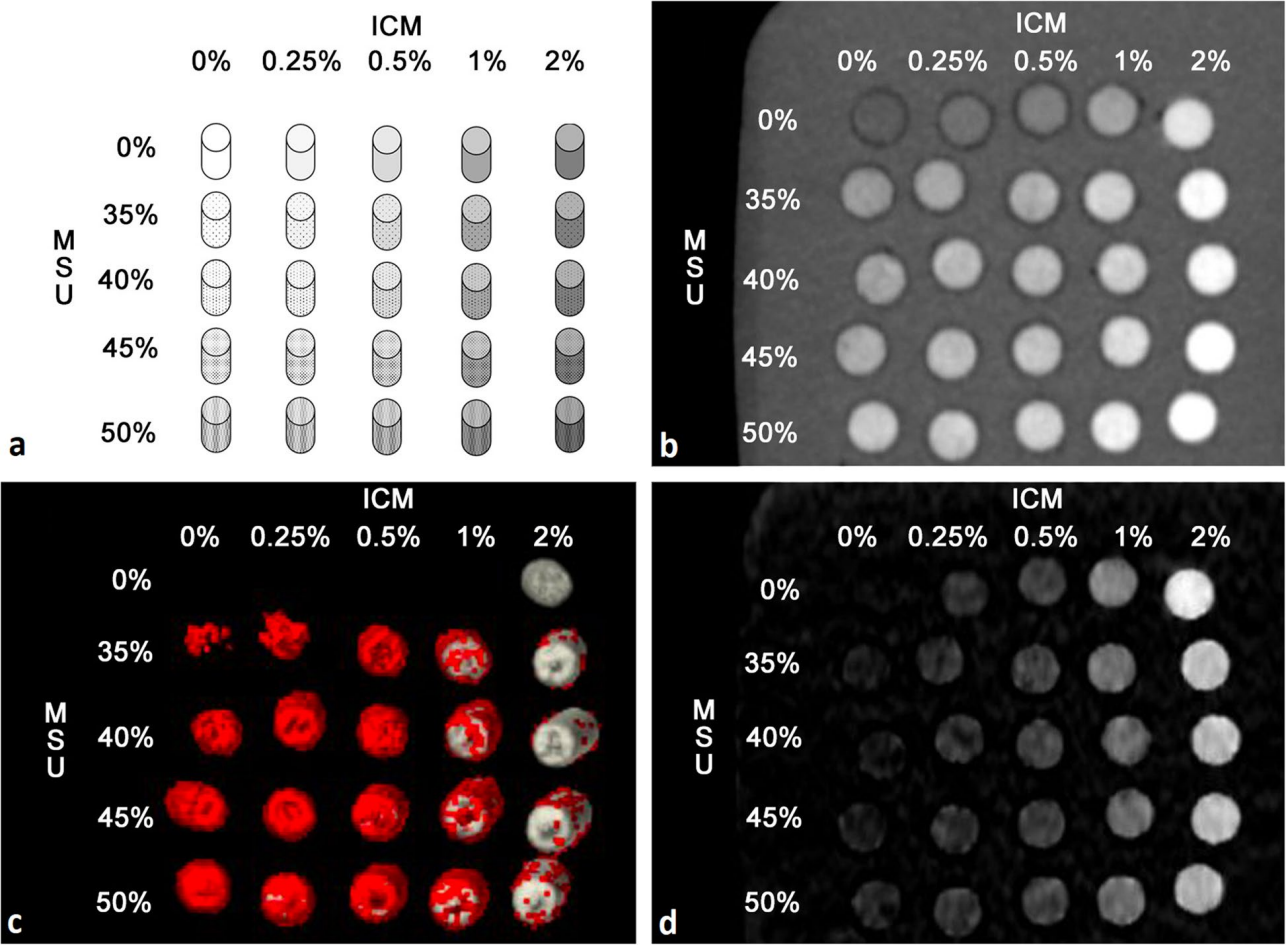
Grid phantom. All 25 syringes were arranged in a grid-like pattern ordered by the concentration of uric acid (MSU) and iodinated contrast medium (ICM) and surrounded by water. **a** Scheme of the grid phantom. **b** 80-kVp scan (coronal plane). **c** MSU map (detected MSU is marked red). **d** Iodine map (coronal plane)

**Fig. 2:**
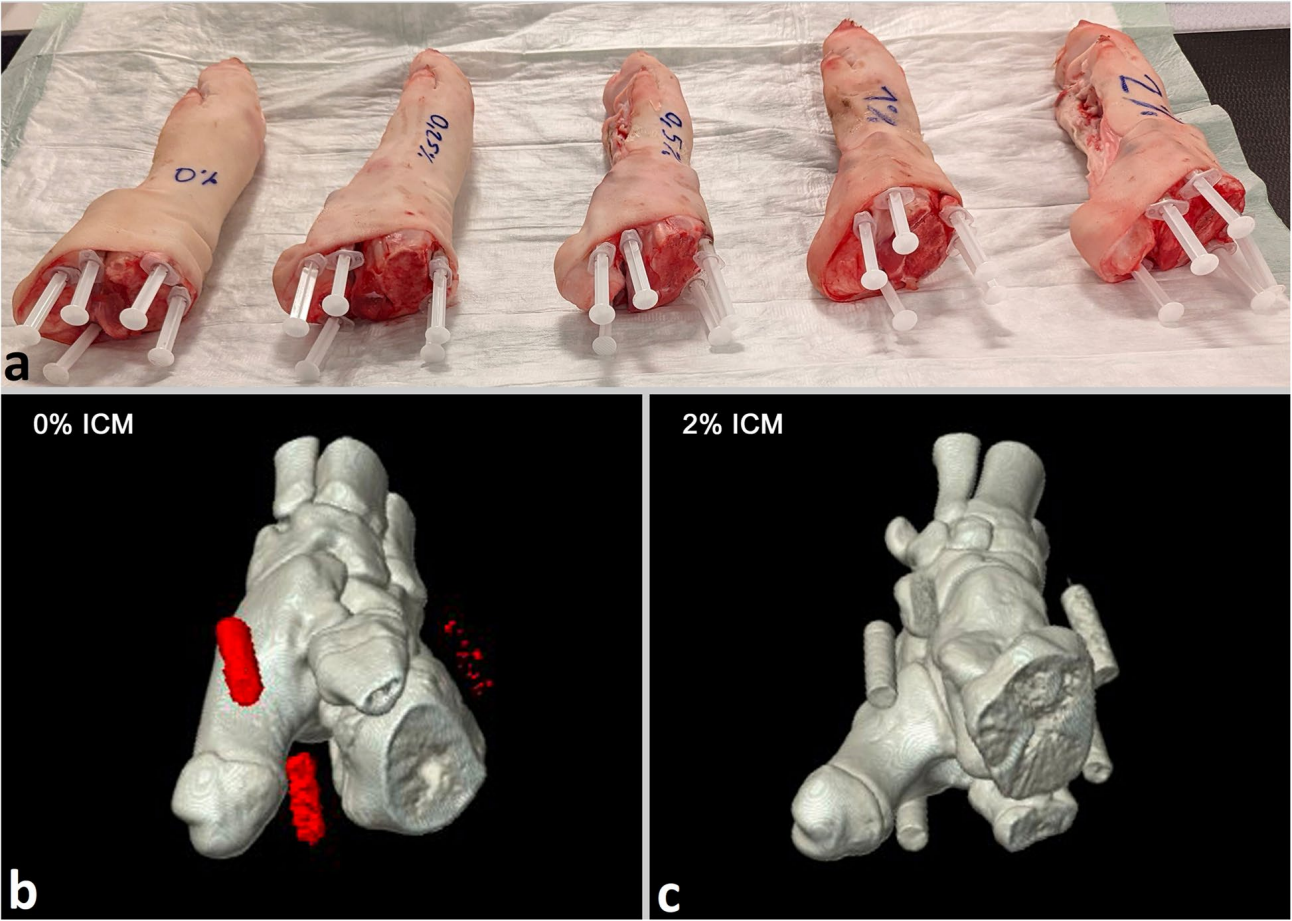
Porcine biophantoms. Five syringes of the same concentration of iodinated contrast medium (ICM) were placed around the elbow joint of each porcine foreleg. They were ordered by their monosodium urate (MSU) concentration in a clockwise direction. **a** Photograph of the five prepared porcine phantoms. **b** Three-dimensional reconstruction of MSU map of the porcine phantom with 0% ICM concentration in the surrounding syringes showing true-positive MSU detections (red) in the syringes with 40, 45, and 50% MSU. **c** No MSU detections are observed with 2% ICM concentration in the surrounding syringes. Instead, the two-material decomposition algorithm assigns the contents of the syringes to calcium/bone (gray)

As a model for gouty tophi, we prepared 25 different suspensions containing MSU (linear formula: ${C}_{5}{H}_{3}{N}_{4}{O}_{3}Na$; molecular weight of 190.09; ${Z}_{eff} 7.7$; Sigma Aldrich, St. Louis, MO, USA) ultrasound gel and an ICM with 370-mg iodine/ml (ULTRAVIST 370, iopromide; linear formula: ${C}_{18}{H}_{24}{I}_{3}{N}_{3}{O}_{8}$; molecular weight of 791.1; ${Z}_{eff} 41.4$; Bayer Vital GmbH, Leverkusen, Germany) in various concentrations. MSU concentrations of 0, 35, 40, 45, and 50% were selected based on previous work [12–16]. The suspensions were prepared with a laboratory precision scale, protocoling the exact MSU mass of each sample, and filled in syringes. The syringes were placed in a grid-like arrangement ordered by MSU and ICM concentration and surrounded by water (Fig. 1). Five porcine forelegs, fresh from the slaughterhouse, were prepared as realistic biological phantoms with bone, muscle, and cutaneous and subcutaneous tissues. Five different syringes containing MSU in ascending order of the same ICM concentration were placed around the elbow joint of each porcine foreleg. Thus, gouty tophi were simulated in an unenhanced and four different contrast enhancement situations (Fig. 2).

### Scan protocol, dose parameters, and image reconstruction

The grid phantom and the five porcine phantoms were separately scanned in a 320-row DECT scanner (Aquilion ONE Vision Edition, Canon Medical Systems, Otawara, Japan). Ten DECT datasets were acquired per phantom using a single-source technique with acquisition of two sequential single-source volumes at 80 kVp and 135 kVp with 16-cm *z*-axis coverage. The scans were acquired without table movement at a tube rotation time of 0.275 s while applying an ascending order of tube currents ranging from 60 to 800 mA at 80 kVp and 10 to 140 mA at 135 kVp (Table 1). All scans were reconstructed using adaptive iterative dose reduction at a strong iteration level (AIDR3D) with a medium soft tissue kernel (FC13), 0.5-mm slice thickness, and 0.25-mm reconstruction interval. Tube current–time product, computed tomography dose index, and dose-length product were recorded, and the estimated effective dose was calculated using a conversion coefficient for upper extremities of $0.0008\ \frac{mSv}{mGy \times cm}$ [17, 18].

**Table 1:** Acquisition parameters and resulting dose parameters

I (mA) at 80 kVp	I (mA) at 135 kVp	Q (mAs) at 80 kVp	Q (mAs) at 135 kVp	CTDI (mGy)	DLP (mGy × cm)	EED (mSv)
60	10	16.5	2.75	1	16.1	0.013
90	15	24.75	4.125	1.5	24.1	0.019
110	20	30.25	5.5	1.9	30.5	0.024
170	30	46.75	8.25	2.9	46.6	0.037
230	40	63.25	11	3.9	62.6	0.05
290	50	79.75	13.75	4.9	78.7	0.063
400	70	110	19.25	6.8	109.2	0.087
510	90	140.25	24.75	8.7	139.7	0.112
630	110	173.25	30.25	10.7	171.8	0.137
800	140	220	38.5	13.6	218.4	0.175

*CTDI* Computed tomography dose index, *DLP* Dose-length product, *EED* Estimated effective dose (using a conversion coefficient of 0.0008 for upper extremities), *I* Tube current, *Q* Tube current–time product

### Quantitative analysis

All 60 phantom scans (10 grid phantom scans and 50 porcine phantom scans) were analyzed using two-material decomposition for the detection of MSU with clinical standard settings in a proprietary clinical software package (Version 6, Canon Medical Systems, Otawara, Japan). A voxel was assigned to MSU when the calculated dual-energy gradient (attenuation at 80 kVP and 135 kV) was appropriate and a given attenuation threshold exceeded to separate MSU from soft tissues. For each specimen, MSU volumes and MSU masses were estimated using an established method that includes the tophus density into the calculation. MSU detection outside the syringes was classified as “false positive” and not included in the analysis.

For better comparison of MSU detectability, a detection index (DI) in percent was calculated for each syringe by setting the estimated detected MSU mass in relation to the known true MSU mass inside each syringe that has been measured during phantom production:

$$Detection\ index\ [\%]=\frac{detected\ MSU\ mass\ [mg]}{true\ MSU\ mass\ [mg]}\times 100$$

To determine if there were significant differences in the detection index values between the groups, we performed a one-way analysis of variance (ANOVA) with Greenhouse–Geisser correction using Prism (Version 7, GraphPad, San Diego, CA, USA). The ANOVA was conducted with a significance level of alpha = 0.05. We then conducted post hoc tests using Tukey’s multiple comparisons test to determine which groups differed significantly from each other. Data analysis, calculations, and display were performed using Microsoft Excel (2016, Microsoft Corporation, Redmond, WA, USA) and Prism (Version 7, GraphPad).

### Clinical illustration

A single-source DECT examination in the same 320-row scanner (Aquilion ONE Vision Edition) with sequential volume acquisition of two different energy datasets (135 and 80 kVp) was performed before and 3 min after intravenous administration of a body-weight-adjusted dose of 80-mL ICM (1 mL/kg ULTRAVIST 370, Bayer Vital GmbH) in a patient with unclear arthritis of the hand. We used our established clinical DECT protocol for scan acquisition and image reconstruction (30 mAs at 135 kVp and 170 mAs at 80 kVp; rotation time 0.275 s; AIDR3D) and performed two-material-decomposition analysis.

## Results

### Phantom model

The phantom model was successfully produced. The masses of MSU and ICM in each sample are listed in Table 2.

**Table 2:** Phantom production protocol

	Mass (g)	0% ICM	0.25% ICM	0.5% ICM	1% ICM	2% ICM
0% MSU	Total mass	2.06	2.08	2.26	2.26	2.22
	MSU mass	0.000	0.000	0.000	0.000	0.000
	ICM mass	0.000	0.005	0.011	0.023	0.044
35% MSU	Total mass	2.51	2.49	2.60	2.46	2.50
	MSU mass	0.879	0.872	0.910	0.861	0.875
	ICM mass	0.000	0.006	0.013	0.025	0.050
40% MSU	Total mass	2.53	2.68	2.68	2.65	2.74
	MSU mass	1.012	1.072	1.072	1.060	1.096
	ICM mass	0.000	0.007	0.013	0.027	0.055
45% MSU	Total mass	2.63	2.72	2.74	2.79	2.74
	MSU mass	1.184	1.224	1.233	1.256	1.233
	ICM mass	0.000	0.007	0.014	0.028	0.055
50% MSU	Total mass	2.75	2.77	2.86	2.77	2.79
	MSU mass	1.375	1.385	1.430	1.385	1.395
	ICM mass	0.000	0.007	0.014	0.028	0.056

Masses of the suspensions of different concentrations of monosodium urate (MSU) and iodinated contrast medium (ICM) in the 25 syringes. Masses are provided in grams for each suspension in total and separately for MSU and ICM

### Scan protocol, dose parameters, and image reconstruction

The tube current–time products ranged from 16.5 to 220.0 mAs at 80 kVp and 2.75 to 38.5 mAs at 135 kVp. All DECT acquisition and dose parameters are compiled in Table 1.

### Quantitative analysis

**Fig. 3:**
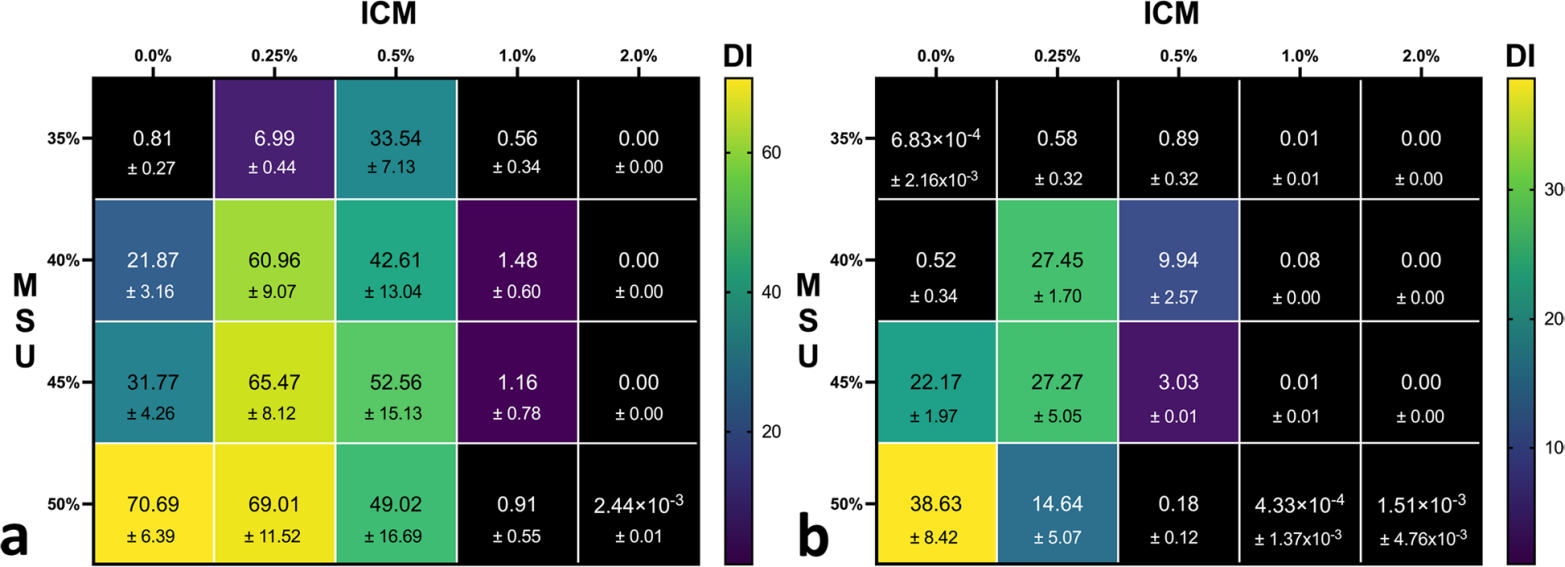
Overview of results for the grid phantom (**a**) and the porcine phantom (**b**). The squares symbolize the suspensions ordered by concentration of iodinated contrast medium (% ICM, *x*-axis) and uric acid (% MSU, *y*-axis). The mean detection index (DI) for each syringe is noted in each square with standard deviation and color coded based on the color bar on the right side

DECT scans of the grid phantom resulted in a higher DI compared to similar scans of the porcine biophantom, independently of the ICM concentration. Per syringe, the DI differed significantly depending on the concentration of MSU and the concentration of ICM in the syringes. The average DI for all four MSU concentrations (35–50%) in the grid phantom scans was 35.4, 54.3, 45.5, 1.1, and 0% (with 0, 0.25, 0.5, 1, and 2% ICM) *versus* 19.4, 17.9, 3.1, 0, and 0%, respectively, in the porcine phantom scans. No false-positive MSU detections occurred, neither when ICM was present in the syringes with 0% MSU, nor in the surrounding bones or soft tissues of the porcine phantoms. Results for both phantoms are presented in Fig. 3 as average DI of all tube currents with standard deviations.

**Fig. 4:**
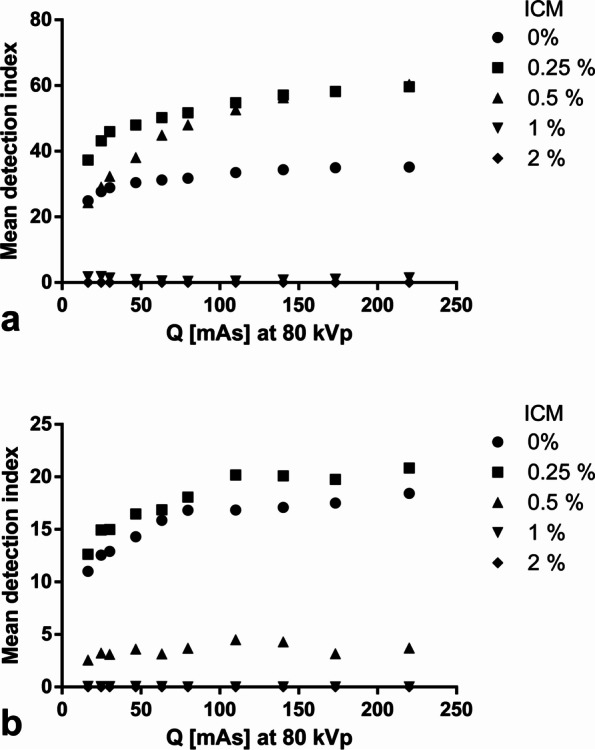
Influence of tube currents in the grid phantom (**a**) and the porcine phantom (**b**). The tube current–time product at 80 kVp (Q; *x*-axis) is plotted against the mean detection index of all monosodium uric acid concentrations of a given iodinated contrast medium (ICM) concentration (legend)

Increasing tube current–time product resulted in higher DI across all MSU probes with ICM concentrations where significant amounts of MSU were detectable (0%, 0.25%, 0.5%) with a saturation effect for very high exposure. For 1 and 2% ICM, there was no effect of tube current–time product on the MSU detection. For details, please see Fig. 4.

The results of the one-way analysis of variance (ANOVA) revealed a significant effect of ICM on DI values in all MSU concentrations across both phantoms (*p* < 0.0001). The Tukey’s test showed mostly significant results (*p* < 0.05) in 75 out of 80 comparisons. In five comparisons, no significant differences were found: 0 *versus* 1% ICM at 35% MSU in the grid phantom and in the porcine phantom for 0 *versus* 1%, 0 *versus* 2% and 1 *versus* 2% ICM at 35% MSU, and 1 *versus* 2% ICM at 50% MSU.

### Clinical illustration

**Fig. 5:**
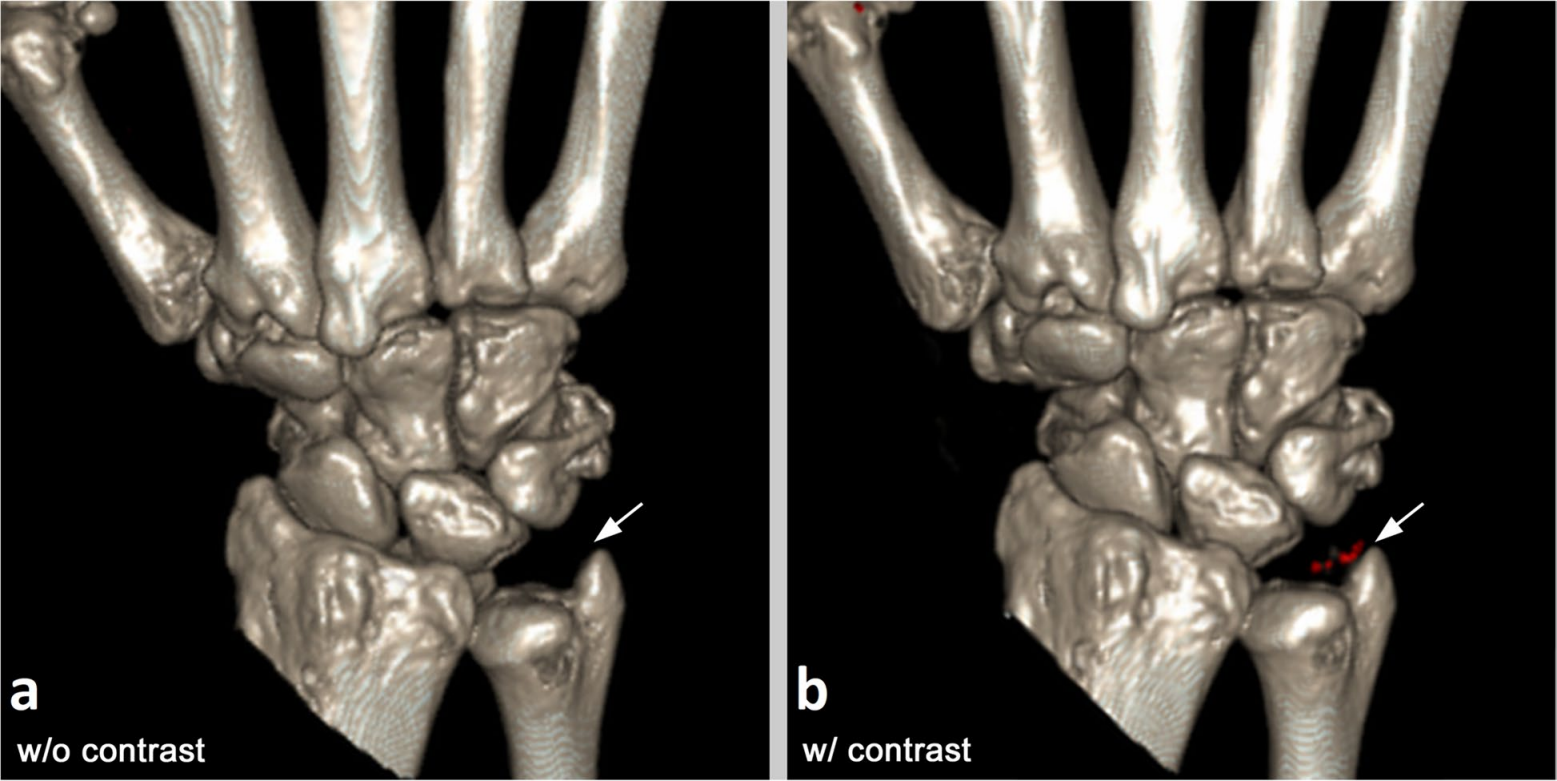
Clinical illustration. Three-dimensional reconstructions of dual-energy computed tomography (DECT) scans before (**a**) and after (**b**) intravenous administration of iodinated contrast medium. Uric acid crystals (red) in the ulnocarpal joint space of the patient are only detected in the contrast-enhanced DECT scan (arrow)

The clinical CT scan showed tophi and synovitis in several regions of the patient’s hand. However, MSU in the ulnocarpal region was only detected after ICM administration (Fig. 5). The total dose-length product of the examination was 93.2 mGy × cm.

## Discussion

We analyzed whether the presence of ICM influences the detection of MSU in DECT, and our findings suggest an effect that depends on the concentrations of ICM and MSU. The rationale for this study were recent developments in DECT with administration of ICM as a diagnostic tool for unclear cases of arthritis with an — to this date — unknown effect of ICM on tophus detection. In our phantom experiments, low ICM concentrations (0.25 and 0.5%) improved detection of low and medium MSU concentrations. However, high ICM concentrations (1 and 2%) significantly hampered MSU detection using a two-material decomposition approach for all MSU concentrations investigated.

The majority of two-material decomposition algorithms available today are primarily developed to differentiate between high-density structures, such as bone and MSU, based on their dual-energy gradient and effective *Z*. However, in clinical practice, accurate identification of these materials can be impeded by image noise and minor tissue changes, such as minimal calcification of the tophus material. To achieve a robust differentiation, software algorithms need to take into account these factors and often display a high level of tolerance towards measurement inaccuracies and mixed tissue compositions. As long as the MSU component remains dominant and the mixed tissue composition does not significantly shift towards the other material, *i.e.*, bone, the algorithm will continue to function optimally. Inorganic calcium with a high atomic number *Z* of 20 is abundant in bone as hydroxyapatite (${Z}_{eff} 15.9$), while MSU crystals resemble an organic purine derivate with a low ${Z}_{eff}$ of 7.7 comparable to soft tissues. Tendons and ligaments have similar ${Z}_{eff}$ and are usually separated from MSU by defining a HU threshold. This threshold is a compromise of sensitivity and specificity, with a high threshold hampering the detection of low-density tophi and low thresholds leading to false-positive findings [15]. Adding ICM with its very high ${Z}_{eff}$ of 41.4 may have two effects: (i) the density (HU) of a voxel increases, and (ii) the dual-energy index changes towards that of iodine. Therefore, comparably low iodine concentrations may increase the density of a tophus with low MSU crystal concentration above the detection threshold while not affecting the dual-energy index enough to hamper MSU characterization in two-material decomposition. This effect might improve the sensitivity of DECT for low-density tophi. However, higher concentrations of contrast medium interfere with proper tophus detection because iodine adds the ${Z}_{eff}$ of MSU within the voxel. With higher ICM concentration, iodine becomes dominant, and the dual-energy gradient of the voxel changes so that the material is no longer characterized as MSU by the two-material decomposition algorithm.

Our experimental findings in phantoms have implications for the applicability of DECT in arthritis imaging. While MRI is no longer the modality of first choice, ultrasound is time-consuming, requires an experienced examiner, and is limited in the evaluation of deeper structures [19, 20]. Standard CT is fast and widely available and allows precise detection of bone erosions; however, DECT offers analysis of different additional aspects beyond the detection of MSU [21–23]. With its ability to detect erosions, bone marrow edema, MSU, and — with ICM — even active soft-tissue inflammation, DECT has the potential to become a powerful diagnostic tool for patients with unclear arthritis [7–9, 24]. However, our phantom study suggests that administration of ICM interferes with the detection of MSU. As sensitivity for MSU varies with the concentration of ICM within the tissue — which for now cannot be predicted — we suggest a reconstruction of two-material decomposition images before and after ICM administration in patients with suspected gouty arthritis undergoing contrast-enhanced DECT until clinical studies confirm that unenhanced DECT is not necessary. In clinical practice, gouty tophi with low MSU concentrations missed by DECT can often only be detected by ultrasound. Since DECT is known to have a limited sensitivity in patients with acute gout and no prior episodes of gouty arthritis, the administration of ICM might help visualize the tophi of low density with high vascularization in these patients [25]. Our findings in a single patient with gouty arthritis indicate that there are MSU tophi that are only detected after ICM administration. Thus, our phantom results and this clinical example indicate that administration of ICM may improve tophus detection due to the limitation of DECT for discrimination of different materials when using two-material decomposition. As for the influence of tube current–time product on MSU detection, our results have validated a nonlinear increasing effect on the DI with a saturation for very high exposure, as described in a previous study [26]. No effect of the tube current–time product has been observed when high ICM concentrations (1 and 2%) were present, as the iodine hampered MSU detection.

Several phantom studies have been performed to investigate detection of MSU with DECT. Recently, Døssing et al. [27] have shown in a phantom setting that DECT can detect MSU, calcium pyrophosphate, and hydroxyapatite crystal deposits and accurately differentiates MSU from CPP and HA crystal deposits. Tse, Kondro, and Kuczynski compared quantification of MSU with DECT in a phantom setting using different decomposition approaches. In this setting, three-material decomposition with virtual monochromatic images detected a lower concentration of MSU than either two-material decomposition or dual-thresholding methods [28].

We are aware that our experimental study has limitations. While we used biological porcine phantom models to create a realistic simulation setting with gouty tophi, the *in vivo* situation may differ. We did not produce multiple copies of the phantoms or repeat measurements with the same tube current for verification. In this study, a specific single-source DECT system was used, so our results may not be transferable to other DECT techniques or scanners from other vendors. Although we took great care to prepare homogeneous specimens, the texture of the crystal suspensions can lead to uneven distribution of MSU and ICM within the syringes. Though our iodine maps allow iodine quantification, this may be not possible in some picture archiving and communication systems depending on the specific software used. Also, quantification of iodine in more or less vascularized gouty tophi might present a challenge in a contrast-enhanced DECT due to unknown cutoff values. We demonstrate the single patient in our study only for illustration of possible clinical relevance. Systematic clinical studies with contrast-enhanced DECT are needed to further analyze this observation.

In conclusion, our phantom study proved that ICM influences the detection of MSU with DECT. While low iodine concentrations improved sensitivity for tophus detection, higher contrast uptake degraded adequate MSU characterization. The clinical impact of our findings has yet to be demonstrated in further studies. For now, the authors recommend adding an unenhanced scan to contrast-enhanced protocols when gouty arthritis is suspected.

## Data Availability

The datasets used and/or analyzed during the current study are available from the corresponding author upon reasonable request.

## Abbreviations

ANOVA: Analysis of variance
CT: Computed tomography
DECT: Dual-energy CT
DI: Detection index
ICM: Iodinated contrast medium
MRI: Magnetic resonance imaging
MSU: Monosodium urate
